# Experimental Study of Aerodynamic Interference Effects for a Suspended Monorail Vehicle–Bridge System Using a Wireless Acquisition System

**DOI:** 10.3390/s21175841

**Published:** 2021-08-30

**Authors:** Yunfeng Zou, Zhipeng Liu, Kang Shi, Shuangmei Ou, Xuhui He, Honggui Deng, Shuai Zhou

**Affiliations:** 1 School of Civil Engineering, Central South University, Changsha 410083, China; yunfengzou@csu.edu.cn (Y.Z.); lzp090700@csu.edu.cn (Z.L.); 204812264@csu.edu.cn (S.O.); xuhuihe@csu.edu.cn (X.H.); 2 National Engineering Laboratory for High-Speed Railway Construction, Changsha 410083, China; 3 School of Civil Engineering, Chongqing University, Chongqing 400045, China; 4 School of Physics and Electronics, Central South University, Changsha 410012, China; xiaoyuj@csu.edu.cn; 5 China Construction Fifth Engineering Division Corp. Ltd., Changsha 410007, China; zhou_shuai@hnu.edu.cn

**Keywords:** aerodynamic interference effects, suspended monorail vehicle–bridge system, crosswinds, wind tunnel test, CFD

## Abstract

The suspended monorail (SM) vehicle–bridge system has been considered a promising modern transit mode due to its clear advantages: low pollution, high safety, convenient construction, and low cost. The wind-induced response can significantly affect the running safety and comfort of this type of vehicle due to its special suspended position from a fixed track. This study is the first to systematically investigate its aerodynamic characteristics and interference effects under various spacing ratios using wind tunnel tests and numerical simulations. A high level of agreement between the wind tunnel test and CFD (computational fluid dynamics) results was obtained, and the aerodynamic interference mechanism can be well explained using the CFD technique from a flow field perspective. A wireless wind pressure acquisition system is proposed to achieve synchronization acquisition for multi wind pressure test taps. The paper confirms that (1) the proposed wireless wind pressure acquisition system performed well; (2) the aerodynamic coefficients of the upstream vehicle and bridge were nearly unchanged for vehicle–bridge combinations with varying spacing ratios; (3) the aerodynamic interference effects were amplified when two vehicles meet, but the effects decrease as the spacing ratio increases; (4) the aerodynamic force coefficients, mean, and root mean square (RMS) wind pressure coefficients for the downstream vehicle and bridge are readily affected by the upstream vehicle; (5) the vortex shedding frequencies of vehicles and bridges can be readily obtained from the lift force spectra, and they decrease as the spacing ratio increases; and (6) a spacing ratio of 3.5 is suggested in the field applications to ensure the running safety and stability of the SM vehicle–bridge system under exposure to crosswinds.

## 1. Introduction

Wind load is considered a typical source of external excitation that intensifies dynamic responses for both bridges and vehicles [1,2,3,4,5,6]. Previous studies have shown that vehicles driving on a bridge can greatly change the aerodynamic characteristics of the bridge when they enter and leave. In turn, the aerodynamic characteristics of a vehicle can also change significantly if the vehicle is in the flow around the bridge [7,8,9]. The aerodynamic forces of bridges and vehicles are larger than those shown in testing models where the vehicles and bridges are separated due to this aerodynamic coupling effect [9]. So far, several train or vehicle overturning accidents caused by strong winds have occurred [10]. Hence, ensuring the running safety and comfort of vehicles during windy conditions has attracted great attention, especially for the development of high-speed railways worldwide [4,11]. Previous studies have systematically investigated the aerodynamic characteristics of the wind–vehicle–bridge (WVB) coupling system through wind tunnel tests, numerical simulation, and theoretical derivation.

For example, Han et al. [7,8] developed an experimental setup to measure the aerodynamic characteristics of vehicles and bridges in a wind tunnel. He et al. [9] experimentally investigated the wind pressure distribution characteristics of a typical high-speed vehicle–bridge coupling system and studied the influences of the wind barrier height and porosity on the aerodynamic characteristics of the vehicle. Xu et al. [12,13] proposed an algorithm to study the dynamic responses of vehicle–bridge coupled systems under exposure to crosswinds. Li et al. [14] followed an analogous approach to analyze the aerodynamic response of railway WVB systems. Some similar theoretical models are also presented in papers published by Xia et al. [15], Olmos and Astiz [16], and Montenegro et al. [17]. Recently, He et al. [18] proposed an efficient analysis framework for high-speed train–bridge coupled vibrations under non-stationary wind excitation based on the pseudo-excitation method (PEM). To reduce the effect of wind on vehicles, wind barriers are installed along bridges. These authors developed an adjustable louver-type wind barrier to intelligently adjust the intensity and angle of incoming winds. They also investigated the aerodynamic characteristics of various vehicle–bridge combinations to explore the effects of wind barriers on the aerodynamic characteristics of a vehicle–bridge system. Later on, they conducted an experimental study to further optimize the parameters of the louver-type wind barrier [19]. Xue et al. [20] studied the aerodynamic force coefficients of a WVB system featuring wind barriers of four different heights and three different ventilation ratios.

Previous studies have mainly been concerned with the conventional vehicle–bridge system where vehicles drive on top of bridges. However, the SM vehicle–bridge system considered herein is significantly different in terms of the positions of driving vehicles and their form [21,22,23,24,25,26,27,28]. Specifically, the driving vehicles are located at the bottom of the bridge structure in the double-line system. The vehicles are always cantilevered on the bridge during operation [26,27,28]. Therefore, SM vehicles are more sensitive to wind load, which gives passengers a significant sense of insecurity [29]. Investigations on the aerodynamic characteristics of the SM vehicle–bridge system are shown in a very limited number of papers. For instance, Bao et al. [29] numerically studied the three-component aerodynamic coefficients and vibration characteristics of the SM WVB coupling system during the meeting of two trains. Some valuable conclusions were drawn in this paper, but this paper was based on a numerical simulation. The results of a detailed wind tunnel test and the wind pressure distribution for the SM vehicle–bridge system were not reported. Moreover, the aerodynamic interference effect between the vehicle and bridge was not well explored.

Additionally, the electronic pressure scanning valve system has been widely used for wind tunnel tests. However, the modules are difficult to measure simultaneously in multi-module practical tests. It is also impossible to apply it to the moving vehicle scale-model, since the data cables create additional resistance, affecting the movement of the vehicle model [30,31]. Additionally, the acquired data have always been terminated in an all-steel closed wind tunnel with an electromagnetic environment. Due to these reasons, the testing precision is decreased to some extent if the electronic pressure scanning valve system is adopted.

To fill this gap, we first studied the aerodynamic characteristics and interference effects of the SM vehicle–bridge system under exposure to crosswinds for various spacing ratios through wind tunnel tests. To address the problem of multi-module synchronization acquisition, a wireless wind pressure acquisition system is proposed. Then, the CFD technique is used to explain the aerodynamic interference mechanism in terms of the wind pressure distribution and flow field features. The rest of this paper is arranged as follows. In Section 2, the experimental background and the test arrangement are introduced. In Section 3, a parametric analysis is carried out for various vehicle–bridge combination conditions. In Section 4, CFD technology is applied to explain the aerodynamic interference mechanism. Conclusions are given at the end of the paper.

## 2. Experimental Background and Arrangement

### 2.1. Experimental Models

Wind tunnel testing was carried out in the high-speed railway wind tunnel test system at Central South University. This is a closed-circuit atmospheric boundary layer wind tunnel with two parallel test sections: the high-speed and low-speed test sections [9]. The study used the high-speed test section, which has a length of 15.0 m, a width of 3.0 m, a height of 3.0 m, and a wind speed ranging from 0 to 94 m/s. The turbulence intensity and unevenness of the flow field were both less than 0.5%.

**Figure 1:**
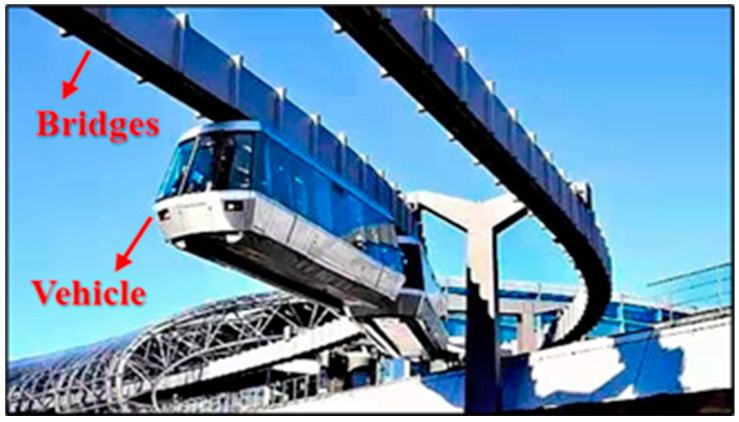
Suspended monorail transit system.

**Figure 2:**
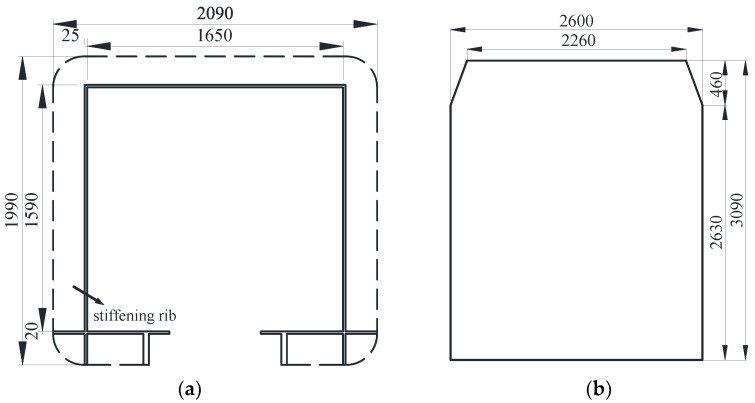
Dimensions of the cross-section: (**a**) Bridge; (**b**) Vehicle (unit: mm).

**Figure 3:**
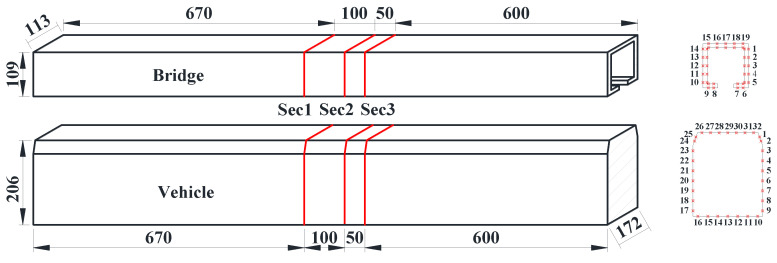
Arrangement of measuring sections and taps (unit: mm).

Typical vehicle–bridge combinations were selected to investigate the aerodynamic interference effect [29,32], as shown in Figure 1. Figure 2 shows the key dimensions of the cross-section for the bridge and vehicle. The geometric scale of the test model was set to 1:15, and the aerodynamic shape was maintained as much as possible with the actual structures. According to the geometric scale and the size of the wind tunnel, the blocking ratio was 4.97%, which satisfies the specification requirement of less than 5%, implying that the effect of the blocking rate can be ignored. For brevity, the details of components with less influence on the aerodynamic shape such as bogies, wheels, and suspension devices were neglected when designing the model [29]. To provide sufficient strength and stiffness, the surfaces of the test models were organic glass, while their skeletons were made from steel pipes. The test models are shown in Figure 3. The width of the bridge model was 1.65 m. To consider the thickness of the steel plate, 50 mm and 40 mm were added to the width and height of the bridge, respectively, as shown in Figure 2a. Hence, the width and height of the scaled bridge model were 113 mm and 109 mm (calculated by (1650 mm + 50 mm)/15 = 113 mm and (1590 mm + 40 mm)/15 = 109 mm).

The experimental flow field was uniform with a wind attack angle of 0°. The test wind speed was set to 12 m/s, and the Reynolds number was defined as 9.23 × 10^4^ when considering the width of the bridge as being the characteristic dimension to prevent local vibrations of the model and ensure the accuracy of the results. Due to the blunt shape of the models adopted herein, the effect of the Reynolds number can be ignored [33]. To ensure two-dimensional flow, the sections were set in the middle of the model, far away from the endplate to avoid the influence of the effects of endplates. Furthermore, the endplates were made of thick planks and rigidly fixed to the test models to prevent local motion. On this basis, three pressure-measuring sections were set up on the bridge and the vehicle models in the length direction. Detailed layouts of the measuring sections and taps for both the bridge and vehicle models are shown in Figure 3. Nineteen pressure measuring taps were arranged equally on both the inside and outside of each pressure measuring section for the bridge model. Thirty-two pressure measuring tapes were placed on the vehicle model. Thus, a total of 420 taps were arranged for the double-line vehicle–bridge system, and all taps could be measured simultaneously with the aid of a wireless acquisition module.

The wind pressure was measured by the proposed wireless acquisition module. A total of eight modules were used in the study. Each module had 64 channels, and the testing accuracy was 0.05%. To confirm the testing accuracy, a reference wind speed was measured using a pitot tube located at the centerline of the test section, about 3 m upstream from the testing models, and the sampling frequency was set as 330 Hz, with each sampling period being 60 s.

### 2.2. Wireless Acquisition System

**Figure 4:**
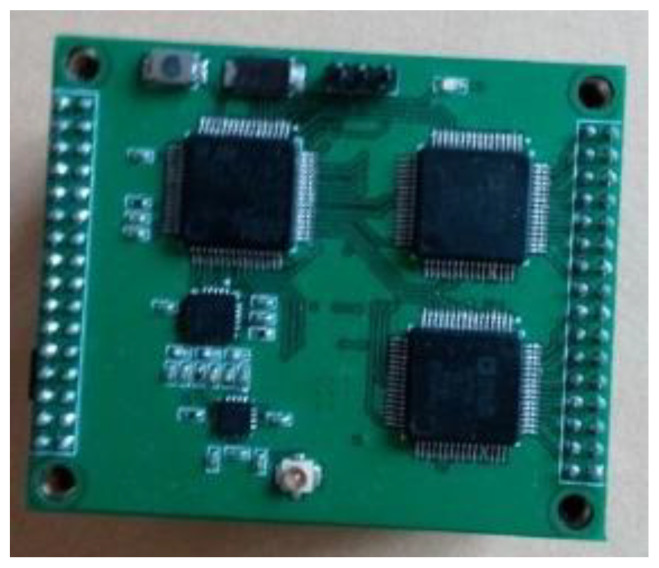
Wireless wind pressure test system developed in the present study.

The system hardware module consists of six parts, namely, an AD converter, single-chip, wireless communication, SD storage system, power management, and USB transceiver [34]. The wireless wind pressure acquisition module is shown in Figure 4.

#### 2.2.1. AD Converter

The input of the air pressure sensor is ±5 V of bipolar voltage, and multiple channels implement synchronous sampling to meet the requirements of the wireless pressure acquisition module. The AD converter is characterized by multichannel synchronous sampling and a bipolar input, and AD7606 was selected as the AD converter for the system. AD7606 is an 8-channel synchronous sampling ADC with a bipolar input, which supports two input options, namely, ±5 and ±10 V, and combines two AD7606 units into a 16-channel synchronous acquisition system with a bipolar input to satisfy the system’s requirements.

#### 2.2.2. Single Chip

To achieve high-speed sampling and control two AD7606 units sufficiently, STM32F405, a Cortex-M4 core (with additional floating-point units and enhanced DSP commands based on a Cortex-M3 core)-based 32-bit single-chip was employed in the wireless acquisition module. In comparison with the previous STM32F1 series, the computing power of the STM32F405 was significantly improved, and is suitable for complex computational and control purposes.

#### 2.2.3. Wireless Communication Module

To ensure stable data transmission in the all-steel closed wind tunnel, the wireless communication system must have strong penetrability and diffraction capacities to send control commands outside the wind tunnel to the data acquisition board. Therefore, the EBYTE E50-TTL-500 wireless transmission module, a wireless serial transmission module within the band range of 148–173.5 MHz, was used, and an RF amplifier was added to the RF front end. The transmitting power and receiving sensitivity can reach 500 mW and −121 dBm, respectively. The air interface user rate (AIUR) of wireless communication is adjustable within the range of 1–25 kbps.

#### 2.2.4. Power Management System

The sensor was powered by a 12 V lithium battery, and the voltage required for the data acquisition board was 5 V/3.3 V. To guarantee long-term stability, the system adopts a switching power supply and TI-TPS54394 power management. The TPS54394 is a dual-channel synchronous step-down switching power supply with an input of 4.5–18 V, an output of 0.76–7 V, and a 90/60 m Ohm built-in FET furnished on the high/low side, which supports a constant current of 3 A in the two channels and satisfies the relevant system requirements.

### 2.3. Testing Cases

**Figure 5:**
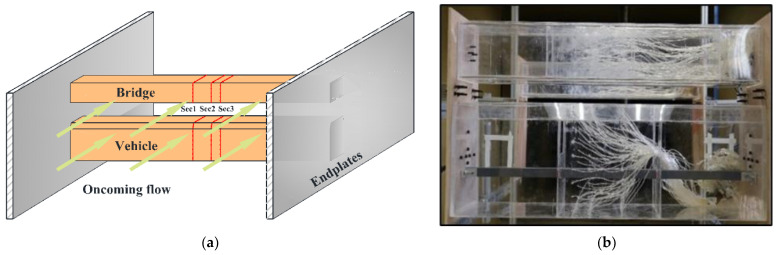
Models tested in the wind tunnel: (**a**) Design drawing; (**b**) Measured drawing.

To study the aerodynamic interference between the bridge and vehicle, the wind pressure for various *D*/*B* ratios was measured. The test cases are listed in Table 1. A schematic diagram of the line spacing is shown in Table 1, where *D* represents the net distance between the upstream and downstream bridges and *B* denotes the width of the bridge. A single-line vehicle–bridge combination was also included to act as a reference for the other cases. Figure 5 shows the testing photos taken in the wind tunnel. From the former studies, it is known that there is a critical value in the range of 2–3 for two parallel square columns. For the downstream column, the aerodynamic force will change greatly when *D*/*B* nears the critical value. In this regard, five *D*/*B* ratios were assessed (1.66, 2, 2.54, 3, and 3.5) with one vehicle. As shown by [33], the aerodynamic interference greatly impacts the downstream box girder. Hence, *D*/*B* ratios of 4, 4.5, and 5 were also added for Cases 3 and 4.

### 2.4. Data Post-Processing

The pressure coefficient *C_pi_*(*t*) is defined as

(1) $$C_{pi}(t)=\frac{P_{i}(t)-P_{0}}{0.5\rho U_{\infty }^{2}}$$

where *P_i_*(*t*) is the wind pressure time history at point *i* measured by the pressure scanning valve, which gives a positive value when the pressure acts into the model surface and vice versa for negative values; *P*_0_ indicates the static pressure for reference; and *ρ* represents the air density, which can be determined using the temperature and atmospheric pressure measured at the beginning of the wind tunnel test. In practice, the value is approximately 1.225 kg/m^3^. *U*_ꝏ_ is the oncoming wind speed at the height of each test model.

The mean value and RMS (root mean square) value of the wind pressure coefficient can be obtained using Equations (2) and (3), respectively:

(2) $$C_{p,mean}=\frac{\sum_{j=1}^{N}C_{p}(j)}{N}$$

(3) $$C_{p,rms}=\sqrt{\frac{\sum_{j=1}^{N}{\left(C_{p}(j)-C_{p,mean}\right)}^{2}}{N-1}}$$

where *C_p_*(*j*) represents the *j*th value of the wind pressure coefficient at the measuring points, and *N* is the number of measuring samples.

To investigate the overall aerodynamic force on the model, three-component aerodynamic force coefficients—the drag coefficient, lift coefficient, and moment coefficient—were obtained by integrating the wind pressure coefficient [9]. The aerodynamic force coefficients can be expressed by both the body and the wind axis coordinate system, but they can be mutually transformed. Thus, only the test results for the body axis coordinate system are given in this paper.

**Figure 6:**
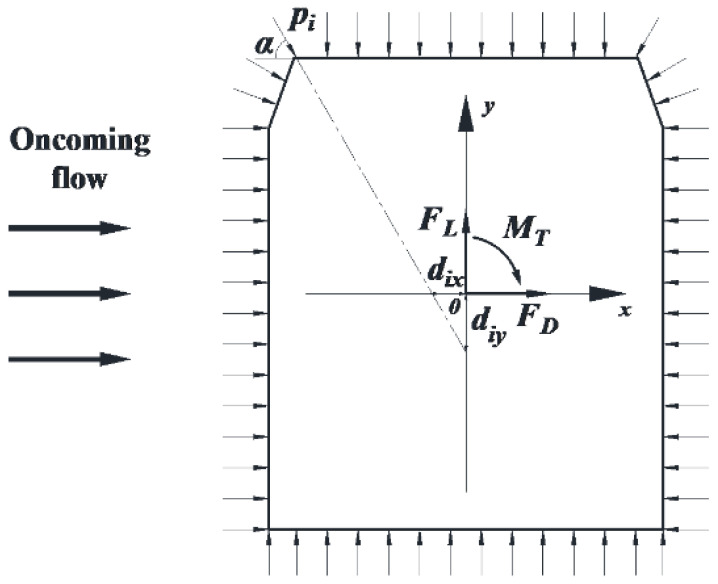
Sketch of the aerodynamic force in the body axis system.

The time histories of the drag coefficient *C_D_*(*t*), lift coefficient *C_L_*(*t*), and moment coefficient *C_M_*(*t*) can be calculated as follows:

(4) $$C_{D}\left(t\right)=\frac{\sum_{i=1}^{n}P_{i}(t)L_{i}\sin(\alpha )}{0.5\rho U^{2}H}$$

(5) $$C_{L}\left(t\right)=\frac{\sum_{i=1}^{n}P_{i}(t)L_{i}\cos(\alpha )}{0.5\rho U^{2}W}$$

(6) $$C_{M}\left(t\right)=\frac{\sum_{i=1}^{n}P_{i}\left(t\right)L_{i}\sin\left(\alpha \right)d_{iy}+\sum_{i=1}^{n}P_{i}(t)L_{i}\cos(\alpha )d_{ix}}{0.5\rho U^{2}W^{2}}$$

where *L_i_* is the characteristic length of the taps; α represents the angle between the plane of the taps and the horizontal plane; *d_ix_* is the horizontal distance and *d_iy_* is the vertical distance from the center of the model to tap *i*; *W* is the width of the model; *H* is the height of the model; *U* is the oncoming wind speed; and *n* is the number of measuring taps on each measuring section with a value of 32 for the vehicle model and 38 for the bridge model. The aerodynamic force and body coordinate system are shown in Figure 6. The three-component aerodynamic coefficients were first calculated using Equations (4)–(6) for each measuring section, and then the coefficients were averaged to obtain the mean values for the following analysis.

Two dimensionless interference factors were defined to estimate the interference effects for the aerodynamic force under various *D/B* ratios:

(7) $$\lambda_{D}=\frac{C_{D}^{a}}{C_{D}^{b}}$$

(8) $$\lambda_{L}=\frac{C_{L}^{a}}{C_{L}^{b}}$$

where *λ_D_* and *λ_L_* denote the aerodynamic interference factors for the drag and lift coefficients, respectively; $C_{D}^{a}$ and $C_{L}^{a}$ are the resulting aerodynamic forces considering the interference effects; and the denominators $C_{D}^{b}$, $C_{L}^{b}$ represent forces without interference effects. Since the moment coefficients of the bridge and vehicle are close to zero, the interference factors of the moment coefficient will be infinite. Thus, the interference factor of the moment coefficient is not discussed in this paper. The results of the single-line vehicle–bridge model (Case 1), as shown in Table 2, were taken as the denominators for the calculation of *λ_D_* and *λ_L_*. Positive values indicate that the test results for the aerodynamic coefficient have the same sign as Case 1 and vice versa.

## 3. Experimental Validation for the Wireless Acquisition System

**Figure 7:**
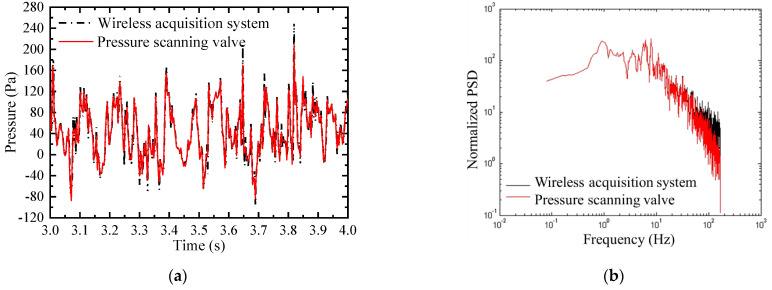
Comparison of wind pressure test results between the wireless acquisition system and pressure scanning valve system in the (**a**) time domain; (**b**) frequency domain.

In this section, the accuracy of the self-designed wireless acquisition system is validated by comparing the test results with measurements from a conventional pressure scanning valve system, the DTC (digital temperature compensation) [9]. The single-vehicle–bridge combination form, Case 1, was used for the comparison. A comparison of the wind pressure test results for the wireless acquisition system and pressure scanning valve system is plotted in Figure 7. There was a high level of agreement between the two testing systems, and the minor difference is negligible in the calculation of three-component coefficients.

The results for the comparison of the three-component coefficient for Case 1 between the wireless acquisition system and pressure scanning valve system are listed in Table 2. There was good agreement between the proposed wireless acquisition system and the pressure scanning valve system, implying that the wireless acquisition system performed well with a high level of precision, satisfying the test requirements in the study.

## 4. Parametric Study

In this section, three typical vehicle–bridge combination forms are systematically discussed to study the aerodynamic characteristics and interference mechanism including a double-line with an upstream vehicle, a double-line with a downstream vehicle, and a double-line with two-vehicle combinations.

### 4.1. Double-Line with an Upstream Vehicle (Case 2)

**Figure 8:**
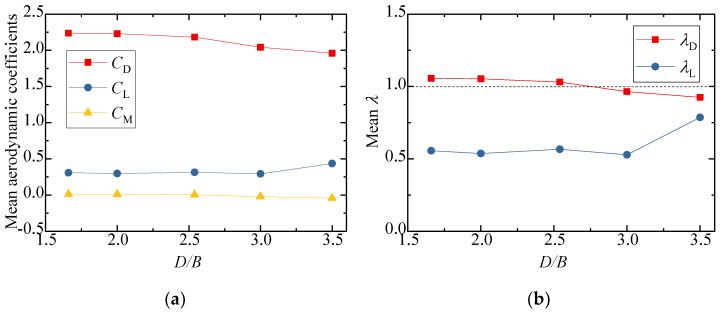
Comparison of the vehicle’s (**a**) aerodynamic coefficients and (**b**) interference factor for various *D/B* ratios in Case 2.

The test results for the aerodynamic coefficients of the vehicle are shown in Figure 8. As can be seen from Figure 8a,b, the drag coefficient decreased slightly with an increase in *D/B*, and the value of $\lambda_{D}$ was close to 1, showing that the interference effect from the downstream bridge is limited. The lift coefficient barely varied, fluctuating around 0.3 when *D/B* ≤ 3.0, but the value increased and $\lambda_{L}$ was close to 1 when *D/B* = 3.5. Figure 8b also shows that $\lambda_{L}$ was always lower than unity, implying that the lift coefficients significantly decreased in comparison to the results for the single-vehicle–bridge mode. The lift coefficients of the vehicle were readily affected by the downstream bridge. The main reason for this is that the negative wind pressure on the top surface of the vehicle increased in the presence of the downstream bridge, resulting in the pressure difference between the top surface AF and bottom surface CD decreasing significantly. Comparably, the moment coefficient was close to null with little change among the different spacing ratios.

**Figure 9:**
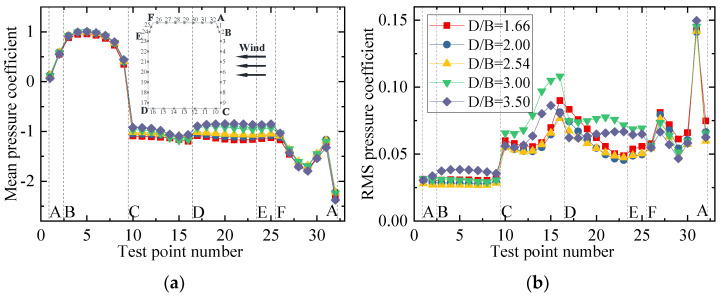
Wind pressure coefficient of the vehicle for various *D/B* ratios studied in Case 2: (**a**) Mean value; (**b**) RMS value.

To show the variation law for the RMS wind pressure coefficient with various spacing ratios, different vertical axis scales were used for the mean and RMS plots in the following studies since the mean values were significantly larger than the RMS values. The results for the wind pressure coefficient of the vehicle are plotted in Figure 9. Figure 9a shows that the mean wind pressure coefficient on surfaces AB, BC, and AF were essentially unchanged for various spacing ratios. However, the mean wind pressure coefficient on the surfaces on CD, DE, and EF was negative and decreased gradually as the spacing ratio increased. The results indicate that the decrease in the vehicle’s drag coefficient was due to the decrease in negative pressure on the leeward side. The increase in the vehicle’s lift coefficient was mainly due to the decrease in the negative pressure on surface CD. For the RMS wind pressure coefficient, when *D/B* ≤ 2.54, the RMS value was unchanged, while it increased greatly on surfaces CD, DE, and EF when *D/B* = 3.00. Additionally, it can be seen from Figure 9 that an obvious fluctuation occurred at point 31, which was attributed to the fact that the oncoming flow was separated at point 32, and point 31 was located in the following wake. This resulted in a significant fluctuation in the RMS wind pressure at point 31.

**Figure 10:**
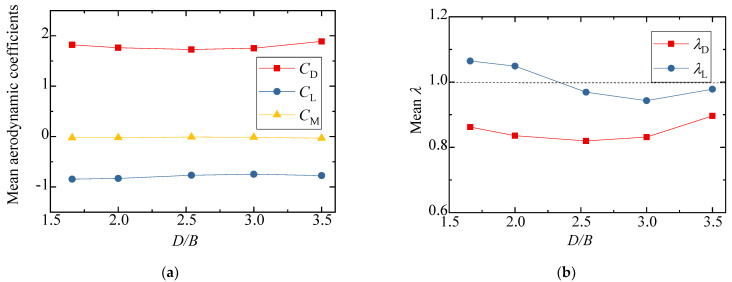
Comparison of the (**a**) aerodynamic coefficients and (**b**) interference factor for the upstream bridge for the various *D/B* ratios investigated in Case 2.

Figure 10 shows the results of the three-component coefficients of the upstream bridge. The drag, lift, and moment coefficients increased slightly as the spacing ratio increased, and the value generally changed a little under different spacing ratios. The drag, lift, and moment coefficients fluctuated around 1.8, −0.8, and 0, respectively. As can be seen from Figure 10b, the interference factor $\lambda_{L}$ was larger than unity when *D/B* ≤ 2.0 and less than unity when *D/B* ≥ 2.5. The interference factor $\lambda_{D}$ was always less than unity for various *D/B* ratios. To this point, the aerodynamic interference effect was shown to mainly affect the lift force, but the effect was weakened with a larger *D/B* ratio.

**Figure 11:**
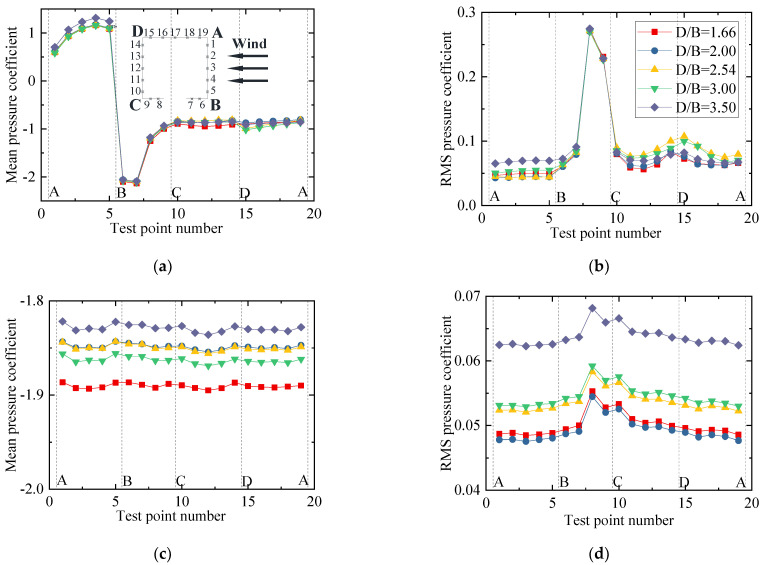
Wind pressure coefficients of the upstream bridge for Case 2: (**a**) Mean values for the outer taps; (**b**) RMS values for the outer taps; (**c**) Mean values for the inner taps; (**d**) RMS values for the inner taps.

The results for the wind pressure coefficient of the upstream bridge are shown in Figure 11. Considering the huge difference in the wind pressure coefficient, the wind pressure coefficients of the bridge for the outer and inner taps are discussed separately to highlight the variation law for the inner taps under various spacing ratios. It can be observed that the variation law for the mean wind pressure coefficient of the outer taps was almost the same under different spacing ratios. However, the RMS wind pressure coefficient of each outer tap was not obviously affected by the spacing ratio. The RMS wind pressure coefficients at points 7 and 8 were maximal, about four to five times the values at other measuring points. For the inner taps of the upstream bridge, the wind pressure was always negative and maintained a stable condition. The mean and RMS wind pressure coefficient of the inner taps increased slightly with an increase in *D/B*, but the growth ranges were limited. The mean wind pressure for the inner taps was almost uniform for a given *D/B* ratio. In addition, for test points 7, 8, and 9, the RMS wind pressure coefficients of the inner taps were also basically the same.

**Figure 12:**
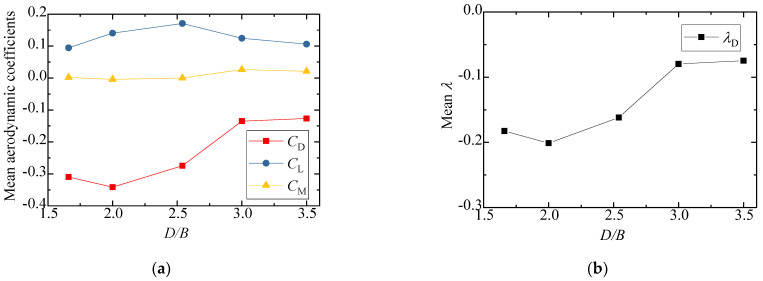
Comparison of the (**a**) aerodynamic coefficients and (**b**) interference factor downstream of the bridge for various *D/B* ratios in Case 2.

Figure 12 shows the test results for the three-component coefficient of the downstream bridge. The results for the single bridge model (Case 1) were used as the denominators for the calculation of the interference factor. Similarly, the interference factor of the lift coefficient is not shown, since the lift coefficient of the single bridge in Case 1 was almost zero. The figure shows that the drag coefficient of the downstream bridge became negative due to the blocking effect of the upstream bridge and vehicle. When *D/B* ≤ 2.00, the drag coefficient decreased slightly as *D/B* increased, and the drag coefficient increased as *D/B* increased when *D/B* > 2.00. The variation law of the drag coefficient interference factor was the same as that of the drag coefficient. Regarding the lift coefficient, it increased as *D/B* increased when *D/B* < 2.50, but when *D/B* > 2.50, the lift coefficient decreased as *D/B* increased. The moment coefficient was close to zero under different spacing ratios.

**Figure 13:**
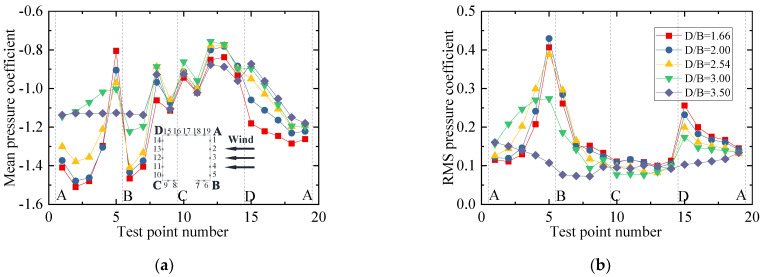

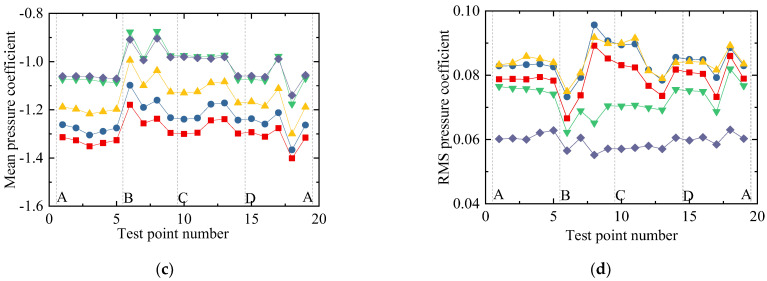
Wind pressure coefficients of the downstream bridge for Case 2: (**a**) Mean values of the outer taps; (**b**) RMS values of the outer taps; (**c**) Mean values of the inner taps; (**d**) RMS values of the inner taps.

The wind pressure coefficient results for the downstream bridge are plotted in Figure 13. It can be seen that the mean and RMS wind pressure coefficients of the downstream bridge differed greatly with the wind pressure distribution of the upstream bridge, as shown in Figure 11. There was an evident fluctuation for the downstream bridge. Due to the shielding effects of the upstream bridge and vehicle, all test taps for the downstream bridge were negative including those on surface AB. Compared with the results shown in Figure 11c and Figure 13c, the wind pressure coefficient of the inner taps for the downstream bridge was more non-stationary than that of the upstream cases, especially for surfaces BC and AD. For both the outer and inner taps, the negative wind pressure decreased as *D/B* increased.

Figure 13b,d show that the RMS wind pressure coefficients for the outer measuring points were basically the same when *D/B* < 2.54, but they decreased significantly when *D/B* > 2.54. For the inner taps, the RMS wind pressure coefficients of most inner points also decreased significantly when *D/B* > 2.54. The RMS wind pressure of the outer and inner taps was stable when *D/B* = 3.5.

**Figure 14:**
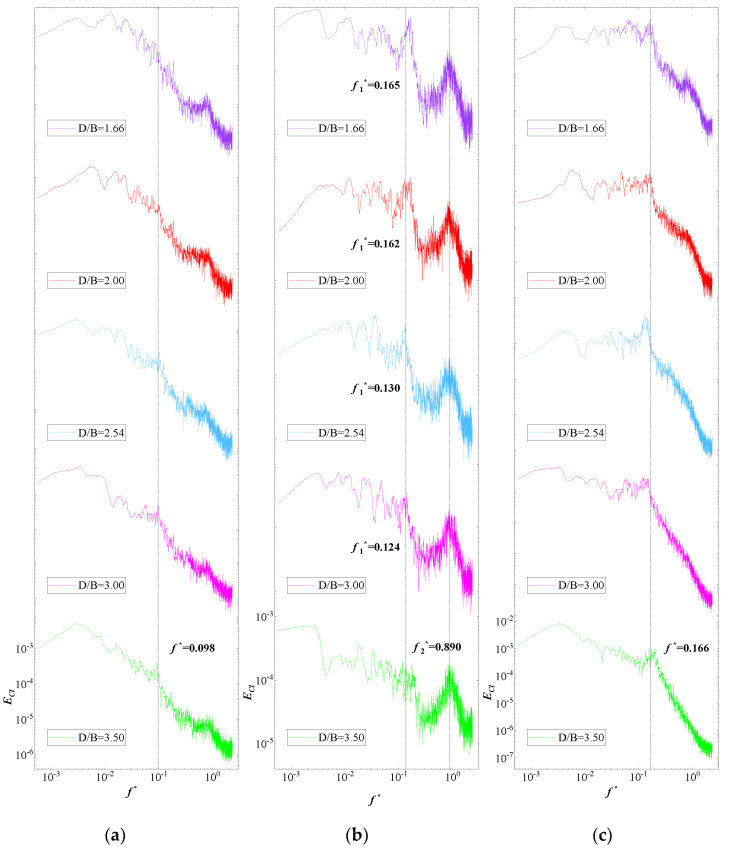
PSD of the lift coefficient for Case 2: (**a**) Vehicle; (**b**) Upstream bridge; (**c**) Downstream bridge.

The power spectrum density (PSD) of the lift coefficients of the vehicle and upstream and downstream bridges for various *D/B* ratios are plotted in Figure 14. A non-dimensional coefficient, the Strouhal number, taken to be St=fH/U where f is the vortex shedding frequency, was used to study the characteristics of vortex shedding [35]. The vortex shedding frequencies can be directly extracted from the peak value from the PSD distribution plot of the lift coefficient. It can be observed that the PSD value of the vehicle was relatively large and stable in the low-frequency domain. In contrast, the distribution of PSD decreased significantly and fluctuated in the high-frequency domain. Additionally, no obvious peak was observed when the ratio of *D/B* was less than 2.0. When *D/B* > 2.0, the peak value was 0.09 Hz. In general, the vortex shedding of the vehicle was relatively weak and the effect of the spacing ratio on the vehicle’s wake shedding was limited.

Figure 14b shows, however, two peak points in the PSD of the upstream bridge, the first at around 0.166 Hz and the second one at 0.890 Hz, which represent the lower and higher vortex shedding frequencies, respectively. Additionally, the lower vortex shedding frequency was found to be relatively unstable. The peak value decreased as the *D/B* ratio increased and it became invisible when *D/B* > 3.00, while the higher value was more stable for all cases. In contrast, only one peak point at around 0.166 Hz was found for the PSD distribution shown in Figure 14c, meaning that there was only a vortex shedding frequency for the downstream bridge. The peak value was basically stable for various *D/B* ratios. However, the vortex frequencies in the high-frequency component were compressed due to the shielding effect of the upstream bridge.

### 4.2. Double-Line with a Downstream Vehicle (Case 3)

**Figure 15:**
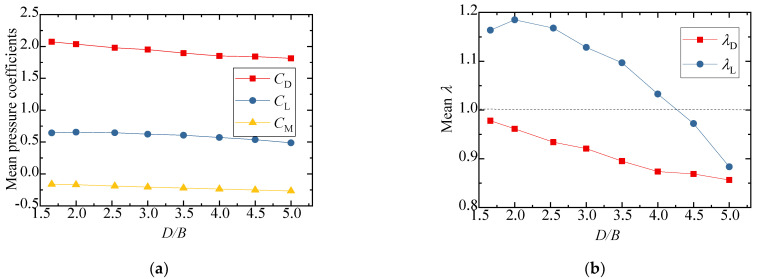
Comparison of the vehicle’s (**a**) aerodynamic coefficients and (**b**) interference factor for the various *D/B* ratios investigated in Case 3.

The results for the three-component aerodynamic coefficients and interference factors are plotted in Figure 15. The results of the single-vehicle–bridge model (Case 1) were taken as the denominators for the calculation of interference factors. It can be concluded that the drag, lift, and moment coefficients decreased slightly as *D/B* increased. Figure 15b shows that the interference factor for drag also decreased as *D/B* increased and the value of $\lambda_{D}$ was always below 1.0, which shows that the interference effect of the upstream bridge weakened gradually and the vehicle aerodynamic drag of Case 3 was less than that of the single-vehicle–bridge model. However, the interference factor of the lift coefficient first increased when *D/B* < 2.0 and then decreased when *D/B* > 2.0. The value of $\lambda_{L}$ reached its maximum with a ratio of 1.2. Additionally, the value of $\lambda_{L}$ was more than 1 when *D/B* < 4.0, while it was less than unity when *D/B* > 4.0.

**Figure 16:**
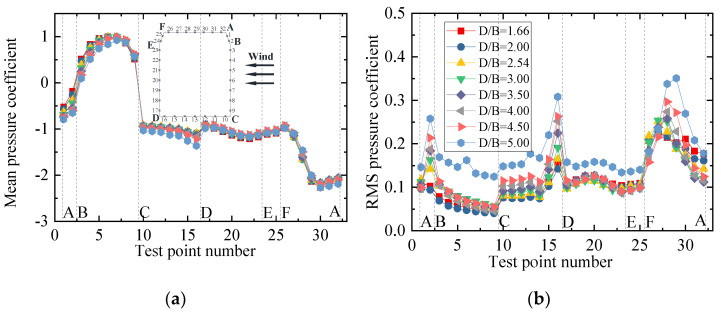
Wind pressure coefficient of the vehicle for Case 3: (**a**) Mean value; (**b**) RMS value.

The mean and RMS wind pressure coefficients of the vehicle under different spacing ratios are shown in Figure 16. It can be seen from Figure 16a that the mean wind pressure coefficient was less affected by the change in *D/B*, and the distribution law of the wind pressure was basically the same for different *D/B* values. The mean wind pressure coefficient on surface BC decreased slightly, which explains the decrease in the vehicle drag coefficient with an increase in the *D/B*. In contrast, the negative wind pressure coefficient on surface CD increased slightly, illustrating why the lift coefficient of the vehicle decreased as the *D/B* increased. For the RMS wind coefficient, the vehicle showed an increasing trend, implying that the wake flow of the upstream bridge became more unstable as the *D/B* increased.

**Figure 17:**
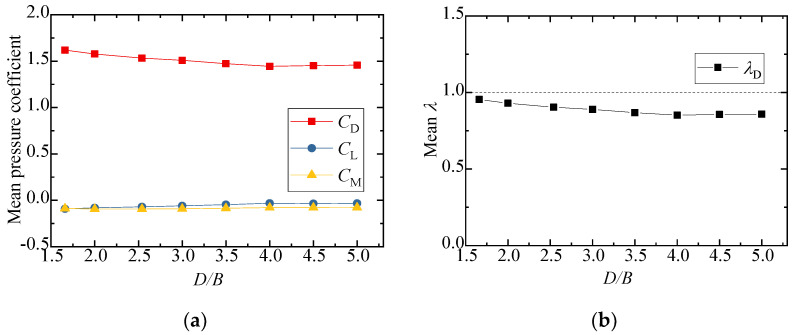
Comparison of the (**a**) aerodynamic coefficients and (**b**) interference factor for the upstream bridge for the various *D/B* ratios investigated in Case 3.

The results of the three-component coefficients of the upstream bridge are plotted in Figure 17. The interference factor of the lift coefficient is not presented in the diagram, since the lift coefficient of the upstream bridge in Case 3 was close to zero. The drag coefficient showed a decreasing trend, while the lift and moment coefficients increased slightly as the *D/B* increased. Generally, the three-component force coefficients of the upstream bridge were less affected by the *D/B* ratio. The interference factor of the drag coefficient was always less than unity, illustrating that the aerodynamic interference of the upstream bridge was weaker and the drag coefficient decreased when the vehicle was located on the leeward side such as in Case 3.

**Figure 18:**
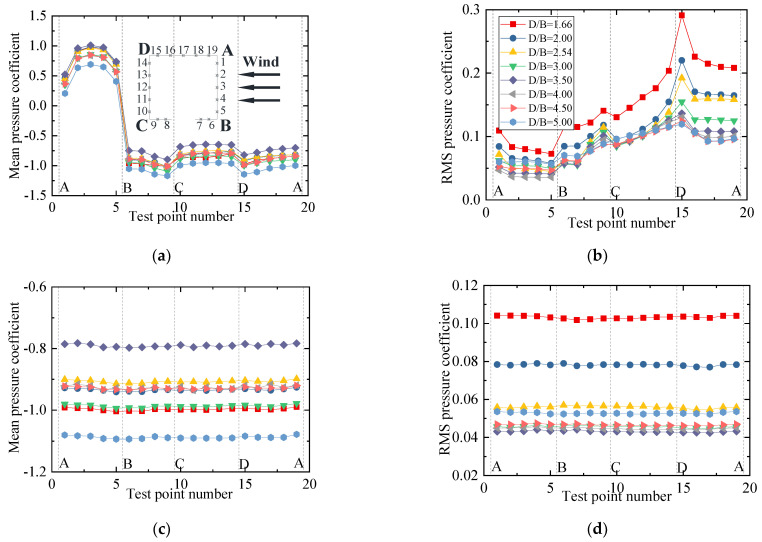
Wind pressure coefficient of the upstream bridge for Case 3: (**a**) Mean values of the outer taps; (**b**) RMS values of the outer taps; (**c**) Mean values of the inner taps; (**d**) RMS values of the inner taps.

Figure 18 shows the mean and RMS wind pressure coefficients of the upstream bridge for various *D/B* ratios. It can be observed that the mean and RMS wind pressure coefficients for all outer measuring points decreased slightly as the *D/B* decreased. However, the distribution of the RMS wind pressure coefficients of the upstream bridge clearly differed from that shown for Case 2. The maximum RMS wind pressure point shifted from point 8 to 15. Figure 18c,d show that the mean and RMS wind pressure coefficients for the inner taps were basically the same for a given *D/B* ratio. However, they decreased as the *D/B* increased. To sum up, the downstream bridge and vehicle can significantly change the flow distribution, affecting the RMS wind pressure of the outer measuring points. The aerodynamic interference effects were found to decrease as the *D/B* ratio increased.

**Figure 19:**
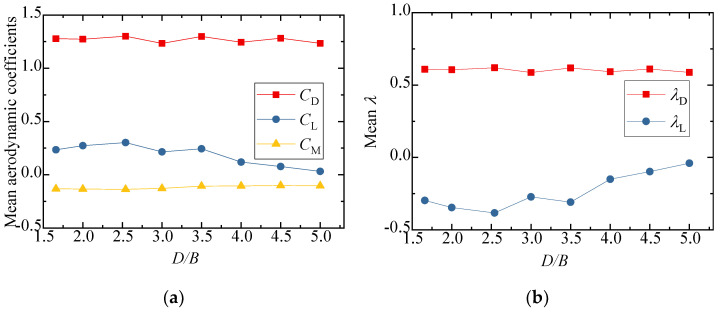
Comparison of the (**a**) aerodynamic coefficients and (**b**) interference factor of the downstream bridge for the various *D/B* ratios investigated in Case 3.

The three-component coefficients of the downstream bridge are plotted in Figure 19. The effect of the *D/B* ratio on the drag and moment coefficients was limited. The drag coefficient barely changed, fluctuating around 1.25, and its interference factor, $\lambda_{D}$, was maintained at 0.6 with an increase in the *D/B* ratio. However, the lift coefficient and lift interference factor were found to be readily affected by the *D/B* ratio. Additionally, the lift coefficient turned negative under the shielding effect of the upstream bridge. However, the interference factors were less than unity, implying that the aerodynamic interference for the downstream bridge was still weaker when the vehicle drove on the leeward side.

**Figure 20:**
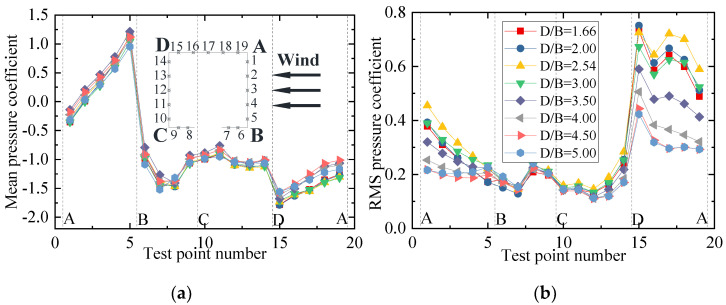

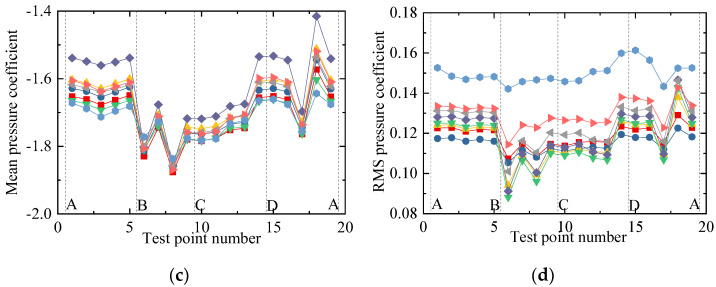
Wind pressure coefficient of the downstream bridge for Case 3: (**a**) Mean values of the outer taps; (**b**) RMS values of the outer taps; (**c**) Mean values of the inner taps; (**d**) RMS values of the inner taps.

Figure 20 shows the mean and RMS wind pressure coefficients of the downstream bridge investigated in Case 3. The mean wind pressure coefficients of the outer taps were basically the same for various *D/B* ratios. Furthermore, the RMS wind pressure evidently fluctuated on surfaces AB and AD. The main reason for this was the effect of the upstream bridge and the immersion of the vehicle into its wake stream. Figure 20c,d show that the negative mean and RMS wind pressure coefficients first decreased when the *D/B* ratio was less than 2.0 and then increased as the *D/B* ratio increased when *D/B* > 2.0. In comparison with the upstream bridge, the mean and RMS wind pressure coefficients of the downstream bridge were found to be more unstable due to the shielding effect.

**Figure 21:**
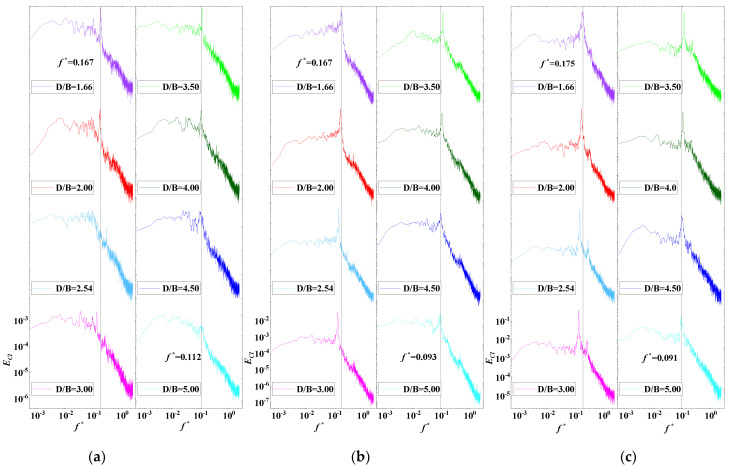
PSD of the lift coefficient for Case 3: (**a**) Vehicle; (**b**) Upstream bridge; (**c**) Downstream bridge.

The PSD value of the lift coefficients for Case 3 is plotted in Figure 21. An obvious peak frequency point was clearly identified in the PSD distributions of the vehicle and upstream and downstream bridges for various *D/B* ratios. However, the frequency point decreased as the *D/B* increased. There was a line to divide the vortex shedding frequency into lower and higher frequencies when *D/B* = 3.00. When *D/B* was no more than 3.00, the vortex shedding frequencies of the vehicle and upstream and downstream bridges were 0.167, 0.167, and 0.175 Hz, respectively. When the *D/B* was larger than 3.00, the vortex shedding frequencies changed to 0.112, 0.093, and 0.091 Hz, correspondingly. Compared with the results for Case 2, the vortex shedding frequency of the vehicle was significantly magnified for Case 3. Unlike for Case 2, a vortex shedding frequency was also presented in the PSD of the upstream bridge. Thus, the aerodynamic characteristics of the vehicle and bridge varied when the vehicle drove on the downstream side.

### 4.3. Double-Line with Two Vehicles (Case 4)

Section 4.1 and Section 4.2 systematically discussed the aerodynamic characteristics of the vehicle and bridge for various *D/B* ratios with one vehicle. To further investigate the aerodynamic interference feature for a suspended vehicle–bridge system, the aerodynamic characteristics of a vehicle and bridge were investigated when two vehicles were driving over it. For brevity, no results are presented for the upstream vehicle and bridge because they are similar to the results of Case 2.

**Figure 22:**
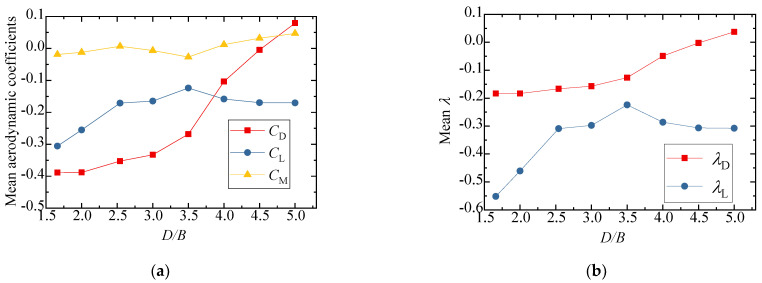
Comparison results for Case 4: (**a**) Aerodynamic coefficients of the downstream vehicle; (**b**) Interference factor of the downstream vehicle.

The three-component coefficients and interference factors of the downstream vehicle with various *D/B* ratios are plotted in Figure 22. Similarly, the three-component coefficient with no interference was taken from the result of Case 1 for the calculation of *λ*. The variation in the aerodynamic coefficients with different *D/B* ratios was different for the downstream vehicles than for the cases with one vehicle. As shown in Figure 22a, the mean lift and drag aerodynamic coefficients turned negative, and the negative drag coefficients decreased significantly as the *D/B* ratio increased. The absolute value of the interference factor was always less than unity, indicating that the aerodynamic coefficients decreased during the meeting of two vehicles.

**Figure 23:**
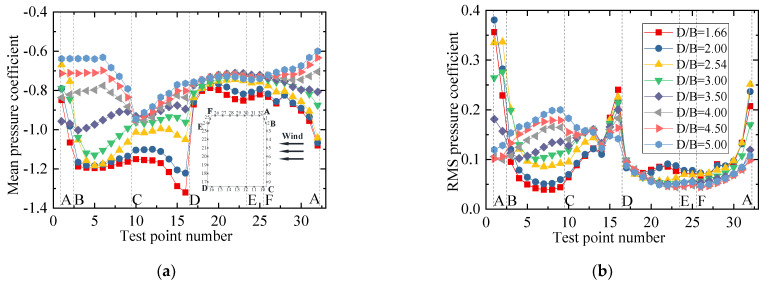
Wind pressure coefficient of Case 4: (**a**) Mean value of the downstream vehicle; (**b**) RMS value of the downstream vehicle.

Figure 23 shows the mean and RMS wind pressure coefficient for the downstream vehicles under various *D/B* ratios. It can be seen from Figure 23a that the mean and RMS wind pressure coefficients of the downstream vehicle were unstable because of the effects of the wake flow from the upstream vehicle and bridge. Compared with Figure 16, the mean wind pressure coefficients were negative for all test points, and the wind pressure on surfaces BC, CD, and AF showed obvious fluctuations. The *D/B* ratio was shown to greatly affect the mean wind pressure of the downstream vehicle, and its negative wind pressure coefficients decreased as the *D/B* increased. In contrast, the effect of the *D/B* ratio was smaller for the upstream vehicle.

**Figure 24:**
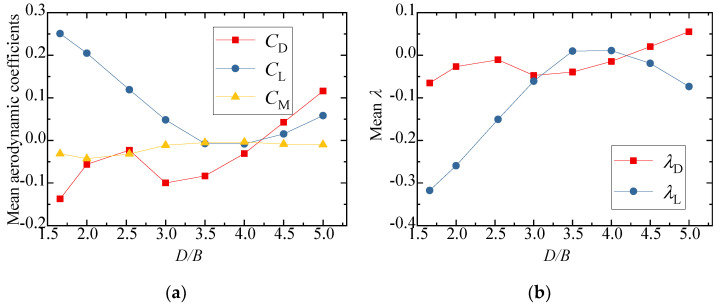
Aerodynamic coefficients and interference factors for Case 4: (**a**) Mean value of the downstream bridge; (**b**) Interference factor for the downstream bridge.

The three-component coefficients and interference factors of the downstream bridges for various *D/B* ratios are plotted in Figure 24. It was found that the aerodynamic coefficients of the downstream bridge showed an obvious fluctuation due to the influence of the wake flow from the upstream vehicle and bridge. The lift coefficients of the downstream bridge first decreased and then increased, and the minimum lift coefficient occurred at *D/B* = 3.5. The drag coefficients of the downstream bridge generally showed an increasing trend as the *D/B* ratio increased. Similar to the downstream vehicle shown in Figure 22b, the interference factor of the downstream bridge was less than 1 and its value was negative.

**Figure 25:**
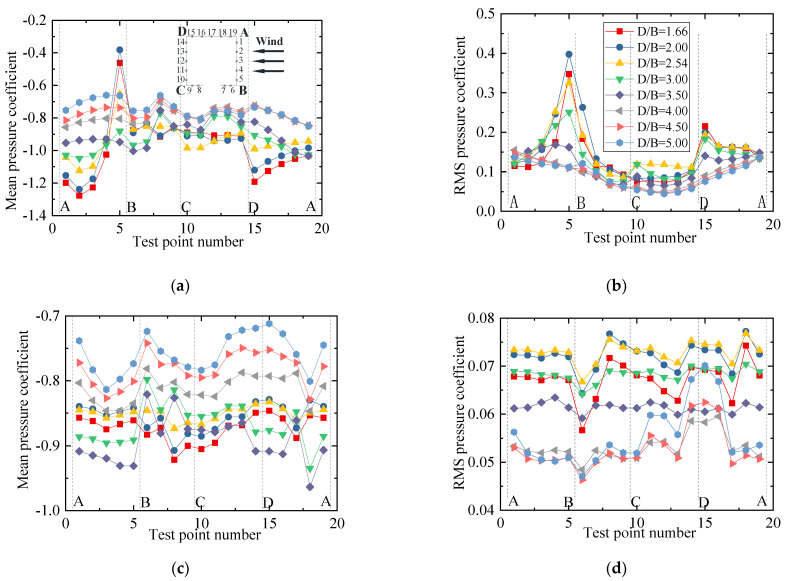
Wind pressure coefficients of the downstream bridge for Case 4: (**a**) Mean values of the outer taps; (**b**) RMS values of the outer taps; (**c**) Mean values of the inner taps; (**d**) RMS values of the inner taps.

The mean and RMS wind pressure coefficients of the downstream bridge for various *D/B* ratios are plotted in Figure 25. Due to the sheltering effects of the upstream vehicle and bridge, the mean and RMS wind pressure distributions of Case 4 are similar to those shown for Case 2. The mean wind pressure coefficients were always negative, and an obvious fluctuating state was found for the inner taps. Figure 25a,b show that the mean and RMS wind pressure coefficients at point 5 were significantly higher than at other measuring points on surface AB when *D/B* ≤ 3.0. The wind pressure coefficients gradually converged without a clear fluctuation when *D/B* > 3.0. Figure 25c,d show that the mean and RMS wind pressure coefficients of the inner taps were greatly affected by the *D/B* ratio. No distinct rule was observed for the inner taps of the downstream bridge. However, the aerodynamic interference effect decreased as the *D/B* increased.

**Figure 26:**
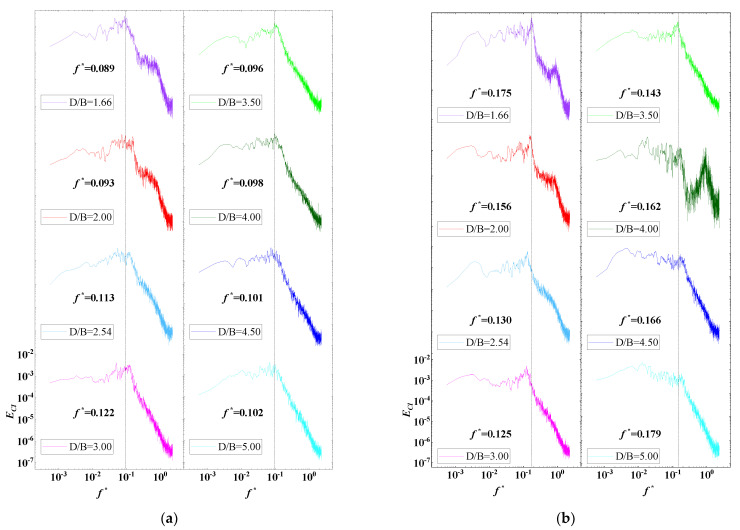
PSD of the lift coefficient for the downstream vehicle under the various *D/B* ratios investigated in Case 4: (**a**) Downstream vehicle; (**b**) Downstream bridge.

The PSD distribution of the lift coefficient for the upstream vehicle and bridge with various *D/B* ratios is plotted in Figure 26. Figure 26a shows a peak in the PSD distribution of the upstream vehicle. Furthermore, the peak frequency was found to decrease as the *D/B* ratio increased, implying that the vortex shedding frequency also decreased when the *D/B* ratio was <3.0. Conversely, the vortex shedding frequency increased as the *D/B* ratio increased when the *D/B* ratio was >3.0. Compared with Case 2, the vortex shedding frequency of Case 4 was greatly increased for the same *D/B* ratio. Thus, the vortex shedding phenomenon was strengthened when considering two vehicles. For the downstream bridge, only two peak frequencies were plotted in the PSD distribution, and the value of the first peak frequency increased obviously when *D/B* = 4.0. Comparably, a peak was found for all other ratios. Compared with the PSD of the downstream vehicle, the peak frequency of the downstream bridge decreased as the *D/B* ratios increased, representing a weakening of the vortex shedding frequencies with a larger *D/B* ratio. Generally, the PSD distribution for Case 4 for the downstream bridges was similar to that shown for Case 2.

## 5. Explanation of the Interference Mechanism

Because the aerodynamic interference effects are mainly reflected during the meeting of two vehicles, the double-line with two vehicles (Case 4) was selected to explain the interference mechanism in this section.

### 5.1. Computational Approach

**Figure 27:**
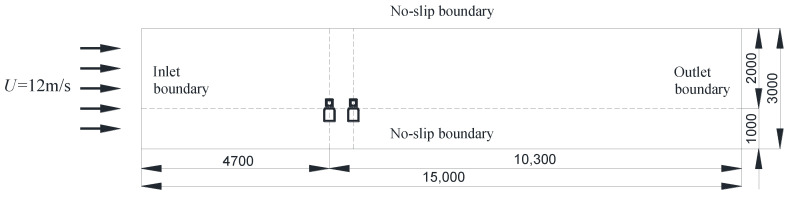
Computational domain and boundary conditions (units: mm).

CFD technology was applied to explain the aerodynamic interference mechanism between a suspended monorail vehicle and a bridge for various *D/B* ratios [36,37,38]. The selected two-dimensional computational domain was consistent with the actual size of the wind tunnel: 3 m × 15 m (height and length). The inlet boundary was a uniform flow with a speed of 12 m/s and the outlet boundary wind speed satisfied the zero-gradient condition. A no-slip condition was applied to the upper and lower boundary layers as well as the surfaces of the vehicles and bridges. Additionally, the Neumann and Dirichlet pressure conditions were utilized at the inlet and outlet, respectively. The computational domain and flow boundary conditions are shown in Figure 27. The turbulence intensity at the inlet was set as 0.5%. The flow was regarded as an unsteady incompressible fluid, since the Mach number of the crosswind was less than 0.3 [39]. An unstructured computing grid was produced using ICEM-CFD software, and an encrypted area was set up to obtain detailed information about the flow field around the vehicle–bridge system. To compare these results with the results of the wind tunnel test, the numerical simulation of the vehicle and bridge was also static. The relative velocity during the meeting of two vehicles was neglected in this study, but it will be the focus of future studies.

Choosing the appropriate size for the simulated mesh was crucial, as this can affect the computational accuracy and efficiency. Therefore, seven grid sizes, 3, 5, 7, 10, 15, 20, and 30 mm were employed. For brevity, no results are presented for this grid independence analysis. It was found that when the minimum mesh size was less than 10 mm, the drag and lift coefficients remained stable. Therefore, the 10 mm mesh was employed in the following studies as the minimum to achieve a trade-off between accuracy and efficiency.

### 5.2. Numerical Verification

**Figure 28:**
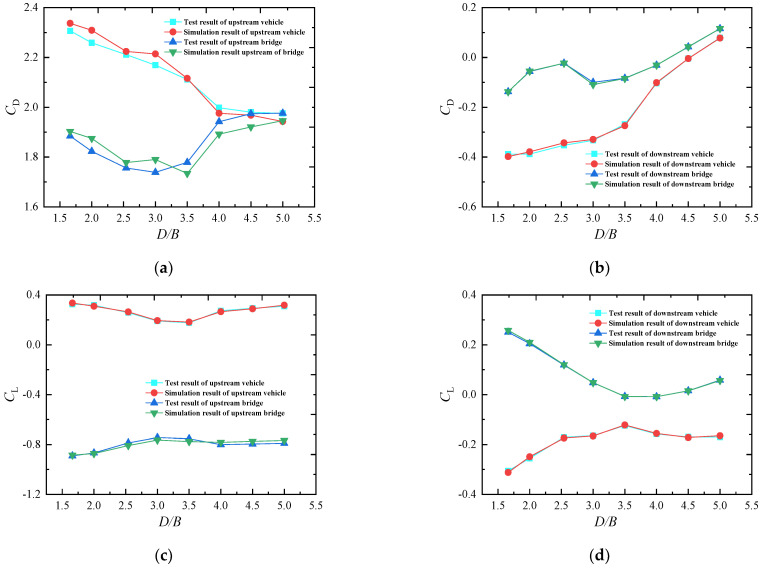
Comparison of the test and simulation results: (**a**) *C_D_* of the upstream vehicle–bridge; (**b**) *C_D_* of the downstream vehicle–bridge; (**c**) *C_L_* of the upstream vehicle–bridge; (**d**) *C_L_* of the downstream vehicle–bridge.

Figure 28 shows a comparison between the test and simulation results for both upstream and downstream trains and bridges with various *D/B* ratios. A high level of agreement was achieved between the two sets of results. The maximum relative error of the bridge was 4.37%, while the maximum value for the vehicle was 3.11%. Both of these are less than the acceptable level of 5%. Thus, the accuracy of the numerical simulation by CFD was validated. The selected grid size and considered boundary conditions are used in the following discussion.

### 5.3. Aerodynamic Interference Mechanism

**Figure 29:**
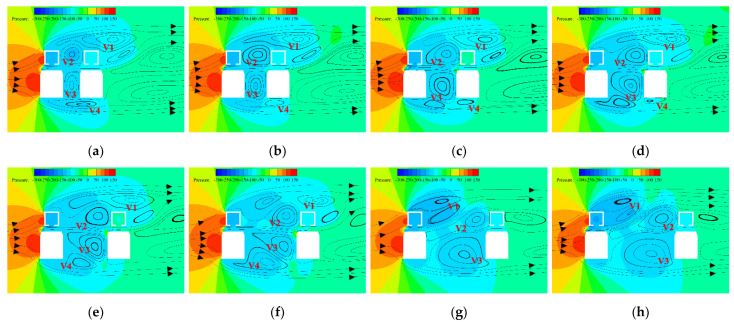
Flow line and pressure nephogram of the vehicle–bridge system investigated in Case 4: (**a**) *D/B* = 1.66; (**b**) *D/B* = 2.00; (**c**) *D/B* = 2.54; (**d**) *D/B* = 3.00; (**e**) *D/B* = 3.50; (**f**) *D/B* = 4.00; (**g**) *D/B* = 4.50; (**h**) *D/B* = 5.00. (units: Pa).

Aerodynamic interference will be discussed from the flow field perspective. The flow line and pressure nephogram of the vehicle–bridge system for various D/B are plotted in Figure 29. As can be seen from the figure, many vortices with different influential radiuses and intensities were produced in the flow field of the vehicle–bridge system, and the vortices were greatly affected by the various spacing ratios. In this regard, the vortices close to the model with high intensities were selected to explain the aerodynamic interference mechanism.

Four main vortices can be observed in the flow field in Figure 29. These are numbered V1, V2, V3, and V4. V1, which was the largest and strongest one, was caused by the separation of the oncoming flow on top of the upstream bridge. When *D/B* ≤ 3, the effective radius and strength of V1 decreased as the *D/B* ratio increased. Moreover, vortex V1 began to split and then moved forward when *D/B* ≥ 3.5. The effect of V1 was larger in the horizontal direction than in the vertical direction, and its shape became longer and flatter. V2 was formed by the backflow between the upstream and downstream bridges. When *D/B* ≤ 3.5, the effective radius and intensity of V2 increased, and the impact height reached its maximum value in the vertical direction, with the shape gradually approaching an ellipse from a circle. When *D/B* ≥ 4, V2 developed gently in the horizontal direction, without another large vortex, greatly influencing the windward side of the downstream bridge. V3, which lay between the upstream and downstream vehicles, was shown to be similar to V2. The variation law of the spacing ratio was similar to that of V2, but the intensity and radius were larger than those of V2, changing the pressure on the leeward side of the upstream vehicle and the windward side of the downstream vehicle. The vortex V4 was produced by backflow on the bottom of the vehicle when the oncoming flow was blocked. V4 was found to be long and oval with a large width in the horizontal direction. When *D/B* ≤ 2.54, the intensity and radius of V4 decreased; when 3 ≤ *D/B* ≤ 4, V4 moved forward. Comparably, the development of V4 was found to be more disordered and the wind pressure influence on the bottom of the upstream and downstream vehicles was weakened. When *D/B* ≥ 4.5, V4 disappeared and there was no large vortex on the bottom of the vehicles.

Figure 29 shows that when *D/B* = 3.5, the upstream bridge line was less affected by vortices. In particular, V2 and V3 moved close to the downstream bridge line, which reduced the negative pressure on the leeward side of the upstream line and greatly reduced the drag in the vehicle–bridge system. Based on the aerodynamic variation law for the vehicle–bridge system under various spacing ratios (Figure 28), it can be concluded that the drag coefficient of the upstream vehicle was small and the drag coefficient of the upstream bridge was also close to the minimum value when *D/B* = 3.5. When *D/B* < 3.5, the drag coefficient of the upstream vehicle and bridge increased greatly. However, the drag coefficient of the upstream vehicle partly decreased, and the drag coefficient of the upstream bridge increased greatly when *D/B* > 3.5. Therefore, the spacing ratio of *D/B* = 3.5 was more beneficial for the running safety of vehicles in terms of the drag coefficient of the upstream rail line. It was also observed that the lift coefficient of the upstream vehicle was the smallest, and the lift coefficient value of the upstream bridge was small when *D/B* = 3.5. Additionally, the downstream rail line was located in the wake area of the upstream one, and the flow velocity was greatly reduced. Furthermore, the aerodynamic force of the downstream rail line was less than that of the upstream one. Hence, a *D/B* ratio of 3.5 is the best choice for field applications in terms of the safety and stability of the whole vehicle–bridge system.

## 6. Conclusions

In this study, the aerodynamic characteristics and interference effects of an SM vehicle–bridge system were studied through wind tunnel tests and numerical simulations. To address the synchronization acquisition for multi-test modules, a wireless wind pressure acquisition system was introduced. First, three typical vehicle–bridge combinations were tested: a double-line with an upstream vehicle, a downstream vehicle, and two vehicles. Then, the aerodynamic characteristics and interference effects were discussed using mean and RMS wind pressure coefficients, aerodynamic coefficients, and the power spectrum density distribution. Finally, CFD technology was used to determine the accuracy of the testing results and explain the aerodynamic interference mechanism. Based on the experimental and numerical results, the following conclusions can be drawn:

- The self-design wireless wind pressure acquisition system shows a high level of precision and can provide a good reference for multi wind pressure test moduli, especially for wind tunnel tests involving moving vehicles.
- Compared with the results for Case 1, the drag coefficients of the vehicle decreased in Cases 2 and 3 as the *D/B* ratio increased, while the effects on the lift and moment coefficients of the vehicles were limited. When considering two vehicles, the aerodynamic interference for the upstream vehicle was amplified, but the aerodynamic interference for the downstream was weakened;
- For all vehicle–bridge combinations, the aerodynamic coefficients of the upstream bridge were less affected by the *D/B* ratio. However, the aerodynamic coefficients of the downstream bridge were significantly affected by the upstream vehicle;
- The mean and RMS wind pressure coefficients for both the bridge and vehicle were unstable due to the effects of wake flow from the upstream vehicle and bridge. The aerodynamic interference of the downstream vehicle and bridge decreased as the *D/B* ratio increased, but the interference effects of the upstream vehicle and bridge were small;
- The peak frequencies were shown in the PSD distributions of the upstream and downstream vehicles. Two peak frequencies were identified in the PSD distribution of the upstream bridge, while only one peak frequency point was indicated in the downstream bridge spectrum. For both vehicles and bridges, the peak frequencies decreased as the *D/B* ratio increased; and
- A *D/B* ratio of 3.5 is recommended to ensure the running safety and stability of the SM vehicle–bridge system in field applications.

Generally speaking, our conclusions are in good agreement with a previously published study [29]; however, the conclusions based on the numerical simulation alone and the geometric shapes of both the bridge and vehicle models are different. In this paper, the studying of aerodynamic characteristics and interference effects for SM vehicle–bridge system was via the wind tunnel test, and the CFD technique was used to explain the interference mechanism for various spacing ratios. The conclusions can provide a good reference for the design of a SM transit system in field applications. Additionally, the proposed wireless acquisition system can maximally solve the issue of synchronization acquisition for multi-test modules, which is beneficial for wind tunnel tests with a large number of measuring points, especially for moving test models.

## Figures and Tables

**Table 1:** Testing cases.

Case No.	Schematic Diagram of Test Models	Description	*D/B*
1	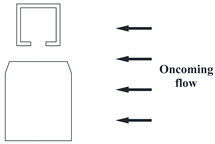	Single-line vehicle–bridge	0
2	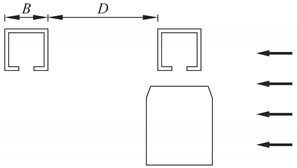	Double-line with upstream vehicle	1.66, 2, 2.54, 3, 3.50
3	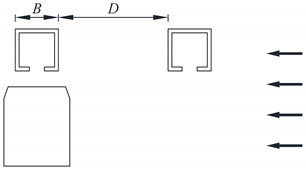	Double-line with downstream vehicle	1.66, 2, 2.54, 3, 3.50, 4, 4.50, 5
4	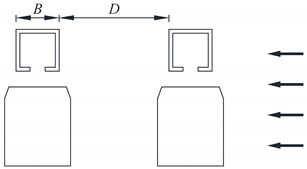	Double-line with two vehicles	1.66, 2, 2.54, 3, 3.50, 4, 4.50, 5

**Table 2:** Three-component coefficients for Case 1.

Results		*C_D_*	*C_L_*	*C_M_*
Pressure scanning valve system	Bridge	2.121	−0.782	−0.031
	Vehicle	2.113	0.551	−0.051
Wireless acquisition system	Bridge	2.193	−0.809	−0.032
	Vehicle	2.069	0.571	−0.048
Relative error	Bridge	3.40%	3.42%	4.27%
	Vehicle	2.11%	3.71%	4.88%

## Data Availability

Not applicable.
